# *S*-Acyl-2-Thioethyl: A Convenient Base-Labile Protecting Group for the Synthesis of siRNAs Containing 5′-Vinylphosphonate

**DOI:** 10.3390/molecules24020225

**Published:** 2019-01-09

**Authors:** Mehran Nikan, Wenyu Li, Garth A. Kinberger, Punit P. Seth, Eric E. Swayze, Thazha P. Prakash

**Affiliations:** Ionis Pharmaceuticals Inc., 2855 Gazelle Ct, Carlsbad, CA 92008, USA; mnikan@ionisph.com (M.N.); lwyt99@yahoo.com (W.L.); gkinberg@yahoo.com (G.A.K.); pseth@ionisph.com (P.P.S.); eswayze@ionisph.com (E.E.S.)

**Keywords:** SATE, siRNA, vinylphosphonate

## Abstract

We recently reported that (*E*)-5′-vinylphosphonate (5′-VP) is a metabolically-stable phosphate mimic for siRNA and demonstrated that 5′-VP improves the potency of the fully modified siRNAs in vivo. Here, we report an alternative synthesis of 5′-VP modified guide strand using *S*-pivaloyl-2-thioethyl (*t*Bu-SATE) protecting group. The *t*Bu-SATE group is readily removed during the final cleavage of the oligonucleotide from the solid support and providing a more convenient route for the synthesis of siRNA guide strand carrying a 5′-vinylphosphonate.

## 1. Introduction

RNA interference (RNAi) is a powerful tool for gene regulation and modulation of disease causing genes [1,2]. Potential of this technology for developing drugs to treat unmet medical needs is proven with recent approval of ONPATTRO™ (patisiran) for the treatment of hereditary transthyretin-mediated amyloidosis. There are several siRNA drugs progressing in the clinic with positive clinical results [3,4,5]. It is necessary to develop stability enhanced siRNA to capture the broader potential of the RNAi therapeutics.

**Figure 1:**
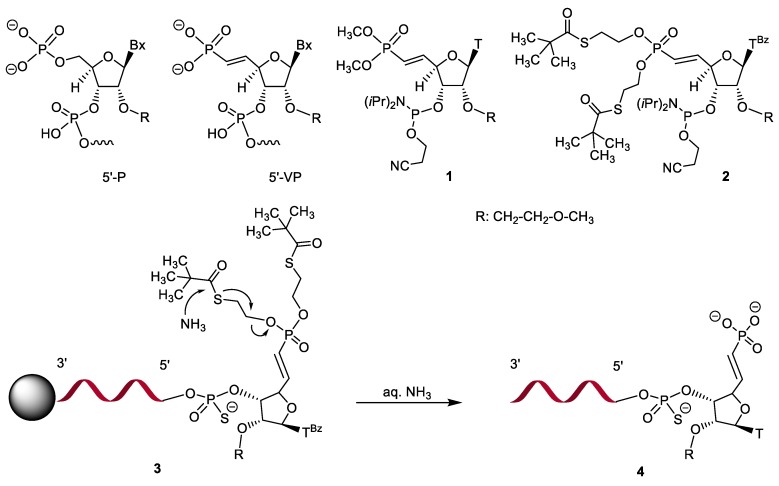
Structure of 5′-phosphate (5′-P), (*E*)-5′-vinylphosphonate (5′-VP), 5′-deoxy-(*E*)-5′-vinyl phosphonate-2′-*O*-(2-methoxyethyl)-thymidine-3′-phosphoramidite bearing methyl **1** and *t*Bu-SATE **2** phosphonate esters; proposed mechanism (**3** and **4)** for aqueous ammonia assisted deprotection of *t*Bu-SATE protecting group.

Judicious placement of nucleic acid modifications in each strand led to the development of siRNA derivatives with both improved potency and stability [6,7,8]. The 5′-phosphate of siRNA guide strand makes critical H-bonding interactions within the Mid domain of the Argonaute-2 (Ago2) that ensures proper positioning and accurate slicing of target mRNA [9,10,11]. The 5′-phosphorylated guide strands are prone to enzymatic cleavage of the terminal phosphate due to the action of phosphatases in vivo [12]. We have previously reported the synthesis of a metabolically stable (*E*)-5′-vinylphosphonate as a phosphate mimic for guide strand (Figure 1, 5′-VP) and its incorporation into the single and double-stranded siRNAs [13]. These modified strands are stable to phosphatase-mediated cleavage and are 5–10 times more potent than the corresponding siRNAs containing a natural phosphate in mice [14]. The X-ray crystal structure of the siRNAs containing 5′-VP in complex with human Ago2 revealed that it maintains the key interactions with Ago2 proteins like the 5′-phosphate moiety [15]. While our reported synthetic strategy was successful in yielding the desired product, further protecting group optimization was required for convenient synthesis of 5′-VP siRNAs. 

In our previous synthesis of 5′-VP siRNA and ss-siRNA, we utilized 5′-deoxy-(*E*)-5′-vinyl (dimethylphosphonate)-2′-*O*-(2-methoxyethyl)-thymidine-3′-phosphoramidite **1** (Figure 1) for incorporation of VP modification at 5′-end of guide strand. The synthesis of siRNAs and ss-siRNA were achieved by standard automated DNA synthesis using the phosphoramidite **1** (Figure 1) and the phosphonate moiety was protected as dimethyl esters [13]. While the methyl groups provided the desired stability during synthesis, their removal required an additional iodotrimethylsilane (TMSI) treatment prior to deprotection of oligonucleotides from the solid support [7]. TMSI mediated deprotection of the methyl groups required strictly anhydrous conditions. Thus, this protecting-group strategy is not amenable to automation and scale-up when a large oligonucleotide library synthesis is intended. These considerations prompted us to explore an alternate synthetic strategy where the phosphonate protecting group can be readily removed under basic conditions. One such approach using pivaloyloxymethyl protecting group was reported very recently [16].

The *tert*-*S*-pivaloyl-2-thioethyl (*t*Bu-SATE) group has been successfully utilized in the synthesis of nucleotide prodrugs and charge neutral oligonucleotides [17,18,19,20]. We envisage that *t*Bu-SATE could be a suitable protecting group for installing the vinylphosphonate during solid phase oligonucleotide synthesis and could be conveniently removed using standard aqueous ammonia deprotection as shown in Figure 1 (**3** and **4**). Herein, we report the synthesis of the 5′-deoxy-(*E*)-5′-vinyl-bis(*t*Bu-SATE)phosphonate)-2′-*O*-(2-methoxyethyl)-thymidine-3′-phosphoramidite **2** (Figure 1) and its application to synthesize 5′-VP modified guide strand of siRNA. Using phosphoramidite **2** the synthesis of 5′-VP modified siRNA was accomplished using conventional oligonucleotide synthesis methods and *t*Bu-SATE protecting group was efficiently removed during aqueous ammonia treatment (**3** and **4**, Figure 1).

## 2. Results and Discussion

### 2.1. Synthesis of 5′-deoxy-(E)-5′-vinyl-bis(tBu-SATE)phosphonate)-2′-O-(2-methoxyethyl)-thymidine-3′-phosphoramidite ***2***

**Scheme 1:**
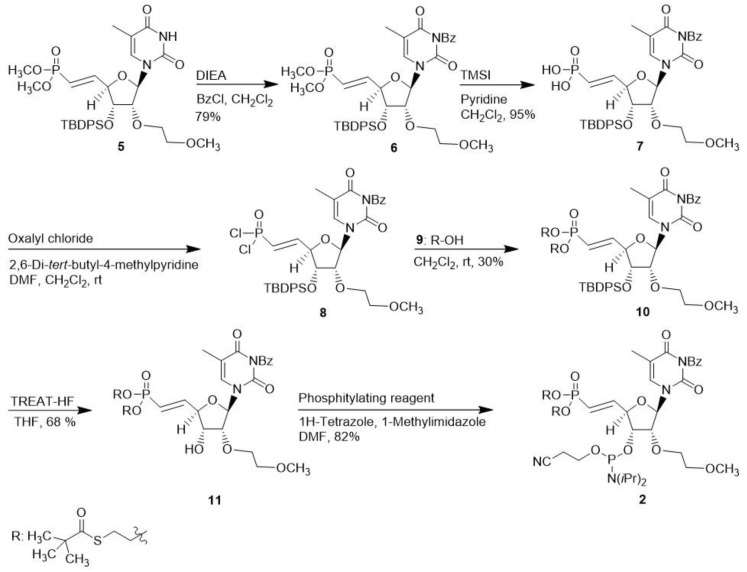
Synthesis of 5′-deoxy-(*E*)-5′-vinyl-bis(*t*Bu-SATE)phosphonate)-2′-*O*-(2-methoxyethyl)-thymidine-3′-phosphoramidite **2.**

Overall strategy for the synthesis of bis(*t*Bu-SATE) protected phosphoramidite **2** is described in Scheme 1. Synthesis started from a known nucleoside **5**, which was synthesized using a reported procedure [13]. First *N*^3^ position of the thymine of compound **5** was protected as the benzoyl amide by treatment with benzoyl chloride (BzCl) in presence of *N*,*N*-diisopropylethylamine (DIEA) in dichloromethane to afford **6** (79%). The methyl protecting groups of **6** were then removed quantitatively by treatment with TMSI in dichloromethane to afford **7**. The compound **7** was first converted to vinylphosphonic dichloride derivative **8** by reacting with oxalyl chloride in dichloromethane containing 2,6-di-*tert*-butyl-4-methylpyridine and catalytic amount of DMF. The vinylphosphonic dichloride derivative **8** was then reacted with *t*Bu-SATE alcohol **9** [20] to afforded bis(*t*Bu-SATE) derivative **10** in 30% yield over two steps. Compound **10** was then treated with triethylamine trihydrofluoride (TREAT-HF) to yield **11** (68%). Finally, **11** was reacted with 2-cyanoethyl-*N*,*N*,*N*′,*N*′-tetraisoproylphosphorodiamidite (Phosphitylating reagent) in the presence of 1*H*-tetrazole and *N*-methylimidazole in DMF to yield compound **2** (82%).

### 2.2. Synthesis of Oligonucleotides Using 5′-deoxy-(E)-5′-vinyl-bis(tBu-SATE)phosphonate)-2′-O-(2-methoxyethyl)-thymidine-3′-phosphoramidite ***2***

**Figure 2:**
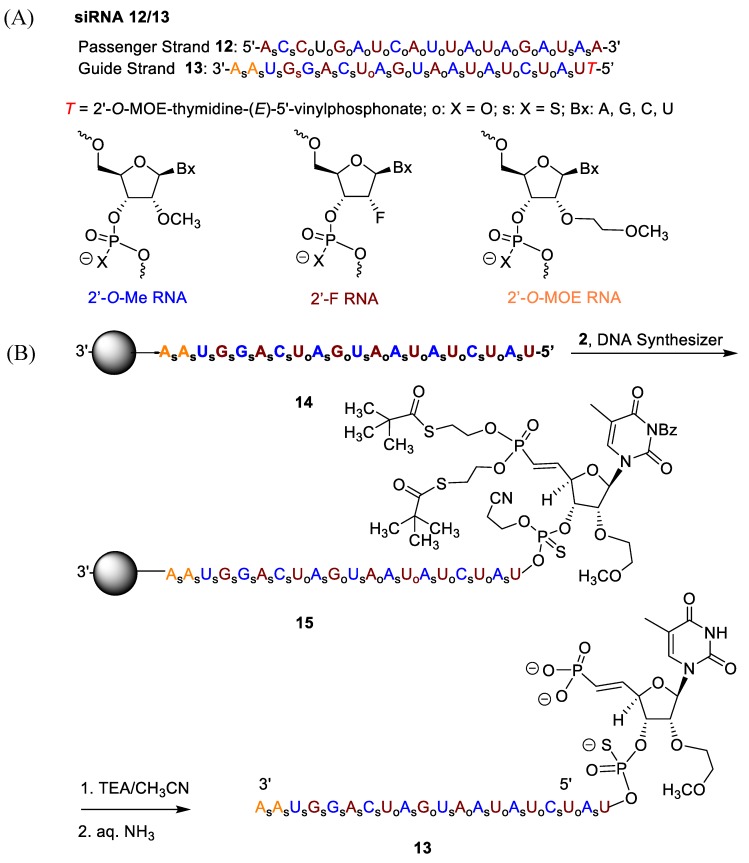
(**A**) siRNA **12**/**13** targeting murine phosphate and tensin homolog (PTEN) containing an alternating 2′-F/2′-*O*-Me motif with a (*E*)-5′-VP-2′-*O*-MOE T; (**B**) Solid-phase synthesis of siRNA guide strand **13** using bis(*t*Bu-SATE)-protected phosphoramidite **2**.

We synthesized a guide strand of previously described modified siRNA **12/13** (Figure 2) targeting murine PTEN containing an alternating 2′-F/2′-*O*-Me motif with a (*E*)-5′-VP-2′-*O*-MOE T [12]. We first synthesized precursor guide strand **14** lacking 5′-VP on a DNA synthesizer using UnyLinker solid support and 2′-F, 2′-*O*-Me and 2′-*O*-MOE modified nucleoside phosphoramidites (Figure 2) [8]. For incorporation of (*E*)-5′-VP-2′-*O*-MOE T residue we used phosphoramidite **2** containing bis(*t*Bu-SATE)-protected 5′-VP (Figure 2) [13]. After the synthesis of guide strand **15** was completed the solid support was treated with 50% triethylamine in acetonitrile containing 1M 2-mercaptoethanol for 30 min to remove the cyanoethyl groups from the phosphate and phosphorothioate linkages. The oligonucleotide was then cleaved and deprotected using aqueous ammonia (28–30 wt%) containing 1M 2-mercaptoethanol at 55 °C for 6 h or at 25 °C for 24 h to yield guide strand **13** (Figure 2). 2-Mercaptoethanol was used to scavenge the acrylonitrile and episulfide (Figure 1) generated during the deprotection of cyanoethyl groups and from *t*Bu-SATE protecting groups respectively. The guide strand **13** was then purified by high pressure liquid chromatography (HPLC) and characterized by LCMS analysis (Table 1).

For comparison we also synthesized guide strand 13 using phosphoramidite **1** containing 5′-VP methyl esters [13]. Synthesis was accomplished on a DNA synthesizer according to the reported procedure [13]. Conventional deprotection condition (aqueous ammonia at 55 °C) was not able to hydrolyze both methyl esters of the 5′-VP moiety. A post-synthetic treatment of solid support bearing guide strand **13** with a solution of TMSI in dichloromethane was needed to completely remove methyl groups. In contrast, for the guide strand **13** containing 5′-VP bis(*t*Bu-SATE) esters, conventional aq. ammonia treatment completely removed *t*Bu-SATE esters from the 5′-VP moiety.

Thus, *t*Bu-SATE approach simplifies the synthesis of 5′-VP modified oligonucleotides and eliminated the need for moisture sensitive and inconvenient TMSI treatment. The overall isolated yield obtained for the synthesis of guide strand **13** using phosphoramidites containing methyl or *t*Bu-SATE esters were similar. Our results suggest that *t*Bu-SATE is a convenient protecting group for the solid-phase synthesis of 5′-VP modified oligonucleotides.

## 3. Materials and Methods

### General

All reactions were carried out under an argon atmosphere using anhydrous solvents unless otherwise stated. Anhydrous solvents and reagents were purchased from commercial sources and used without further purification. Chromatography was performed on Biotage SP1 system equipped with UV detector and SNAP prepacked columns (Biotage LLC, Charlotte, NC, USA). Analytical thin-layer chromatography (TLC) was performed using silica gel 60 F_254_ (Merck KGaA, Darmstadt, Germany) using UV lamp (UVP, Upland, CA, USA) a visualizing agent and developed by a solution of p-anisaldehyde (6 mL), H_2_SO_4_ (8.3 mL), CH_3_COOH (2.5 mL) in C_2_H_5_OH (227 mL) followed by charring. ^1^H-NMR spectra were recorded on a Bruker 300 MHz instrument (Billerica, MA, USA) using residual solvent as reference. ^31^P-NMR spectra are not calibrated and δ is reported relative to 85% phosphoric acid. Yields refer to chromatographically isolated yields. Compound **1** was synthesized using the reported procedure [13].

Compound **2**: To a solution of **11** (0.51 g, 0.651 mmol) in DMF (2.0 mL) were added 1*H*-tetrazole (36 mg, 0.521 mmol), 1-methylimidazole (13 mg, 0.163 mmol), followed by 2-cyanoethyl *N*,*N*,*N*′,*N*′-tetraisopropylphosphordiamidite (0.31 mL, 0.977 mmol). The reaction was complete after 2 h as determined by TLC using 70% ethyl acetate/hexanes as the eluent. The reaction mixture was diluted with saturated NaHCO_3_ and partitioned against ethyl acetate. The aqueous layer was then extracted with ethyl acetate. The ethyl acetate layers were combined, dried over Na_2_SO_4_, filtered and concentrated. The resulting crude compound was purified by silica gel column chromatography and eluted with 70% ethyl acetate/hexanes to yield **2** (0.5 g, 82%). ^31^P-NMR (121 MHz, CDCl_3_): δ 150.54–150.09 (d, 1P), 18.07–17.32 (d, 1P); LR MS (ESI) calcd for C_44_H_66_N_4_O_13_P_2_S_2_ [M − H]^−^ *m/z* = 983.3, found 982.1.

Compound **6**: To a solution of **5** (5.0 g, 7.72 mmol) in CH_2_Cl_2_ (35 mL) were added DIEA (2.69 mL, 15.43 mmol) and benzoylchloride (1.12 mL, 9.65 mmol) and stirred at room temperature overnight. Reaction mixture was concentrated by rotary evaporation under reduced pressure and the residue was purified by silica gel column chromatography by a gradient of hexanes:ethyl acetate (1:1) to ethyl acetate to afford **6** (4.56 g, 79%). ^1^H-NMR (300 MHz, CDCl_3_): δ 7.83–7.95 (m, 2H), 7.58–7.77 (m, 4H), 7.32–7.55 (m, 9H), 6.94 (s, 1H), 6.56–6.77 (m, 1H), 5.79–5.98 (m, 2H), 4.65 (t, 1H), 3.98 (t, 1H), 3.67–3.71 (d, 6H), 3.32–3.61 (m, 5H), 3.29 (s, 3H), 1.9 (s, 3H), 1.01 (s, 9H); ^31^P-NMR (121 MHz, CDCl_3_): δ 19.44; LR MS (ESI) calcd for C_39_H_47_N_2_O_10_PSi [M − H]^−^ *m/z* = 761.2, found 761.3.

Compound **7**: To a solution of **6** (3.82 g, 5.01 mmol) in CH_2_Cl_2_ (15 mL) were added pyridine (1.67 mL, 20.0 mmol) and TMSI (1.28 mL, 12.53 mmol) and stirred at room temperature. Reaction was completed after 30 min as determined by LC-MS (6130 Quadruple LC/MS, Agilent Technologies, Santa Clara, CA, USA). The reaction mixture was concentrated under reduced pressure and the residue was suspended in ethyl acetate and filtered. The filtrate was washed with 0.1 N HCl, dried over MgSO_4_, filtered and concentrated to afford **7** (3.5 g, 95%). ^1^H-NMR (300 MHz, CDCl_3_): δ 7.81–7.94 (m, 2H), 7.56–7.77 (m, 4H), 7.30–7.53 (m, 9H), 6.97 (s, 1H), 6.49–6.72 (m, 1H), 5.89–6.01 (m, 2H), 4.62 (t, 1H), 4.01 (t, 1H), 3.32–3.61 (m, 5H), 3.28 (s, 3H), 1.84 (s, 3H), 1.09 (s, 9H); ^31^P-NMR (121 MHz, CDCl_3_): δ 10.2; LR MS (ESI) calcd for C_37_H_43_N_2_O_10_PSi [M + H]^+^ *m/z* = 735.2, found 735.1.

Compound **10**: To a solution of **7** (2.5 g 3. 41 mmol) in CH_2_Cl_2_ (8.4 mL) were added 2,6-di-*tert*-butyl-4-methylpyridine (4.20 g, 20.48 mmol), oxalyl chloride (0.78 g, 6.15 mmol) and DMF (catalytic). The reaction mixture was stirred at room temperature for 1 h to afford **8** in situ. The reaction mixture was concentrated under reduced pressure and residue dissolved in CH_2_Cl_2_. *t*Bu-SATE alcohol **9** (3.32 g, 20.48 mmol) was added and the reaction mixture was stirred at room temperature overnight. The reaction mixture was concentrated under reduced pressure and the residue was partitioned between ethyl acetate and H_2_O. The organic layer was separated, dried (MgSO_4_), filtered and concentrated. The crude product was purified by silica gel column chromatography (ethyl acetate: hexanes; 1:1) to yield **10** (1.05 g, 31%). ^1^H-NMR (300 MHz, CDCl_3_): δ 7.84–7.95 (m, 2H), 7.59–7.77 (m, 4H), 7.33–7.55 (m, 9H), 6.96 (s, 1H), 6.57–6.78 (m, 1H), 5.80–6.01 (m, 2H), 4.65 (t, 1H), 3.93–4.16 (m, 5H), 3.31–3.65 (m, 5H), 3.29 (s, 3H), 3.10 (m, 4H), 1.90 (s, 3H), 1.23 (s, 18H), 1.10 (s, 9H); ^31^P-NMR (121 MHz, CDCl_3_): δ 17.21; LR MS (ESI) calcd for C_51_H_67_N_2_O_12_PS_2_Si [M − H]^−^ *m/z* = 1021.3, found 1021.4.

Compound **11**: To a solution of **10** (1.0 g, 0.98 mmol) in THF (3.0 mL) was added TREAT-HF (1.2 mL, 7.34 mmol) and stirred at room temperature overnight. The reaction mixture was concentrated under reduced pressure and the residue obtained was purified by silica gel column chromatography using a gradient of ethyl acetate to 50% acetone/CH_2_Cl_2_ to afford **11** (0.52 g, 68%). ^1^H-NMR (300 MHz, DMSO-*d*_6_): δ 7.92–8.04 (m, 2H), 7.75–7.86 (m, 1H), 7.70 (s, 1H), 7.56–7.66 (m, 2H), 6.73–6.95 (m, 1H), 6.00–6.20 (m, 1H), 5.85 (d, 1H), 5.45 (d, 1H), 4.40 (t, 1H), 4.12–4.26 (m, 2H), 3.93–4.10 (m, 4H), 3.60–3.82 (m, 2H), 3.48 (m, 2H), 3.24 (s, 3H), 3.11 (m, 4H), 1.89 (s, 3H), 1.19 (s, 18H); ^31^P-NMR (121 MHz, DMSO-*d*_6_): δ 22.24; LR MS (ESI) calcd for C_35_H_49_N_2_O_12_PS_2_ [M − H]^−^ *m/z* = 783.2, found 783.3.

Compound **13**: The synthesis of modified oligonucleotides was performed on an ABI 394 synthesizer (Biolytic Lab Performance Inc., Fremont, CA, USA) on a 2 µmol scale. A 0.1 M solution of 2′-F, 2′-*O*-Me, and 2′-*O*-MOE, modified nucleoside phosphoramidites in anhydrous acetonitrile (CH_3_CN) and were used for the synthesis. Phosphoramidite **2** was dissolved in 30% dichloromethane in anhydrous CH_3_CN (0.1 M) and used for the oligonucleotide synthesis. The modified oligonucleotides were synthesized on VIMAD UnyLinker^TM^ (AM Chemicals, Oceanside, CA, USA) solid support. Dichloroacetic acid (6%) in toluene was used as detritylating reagent. 4,5-Dicyanoimidazole in the presence of *N*-methylimidazole in CH_3_CN was used as activator during the coupling step. During the coupling step, nine equivalents of phosphoramidite solutions were delivered and the coupling was carry out for 10 min. Phosphorothioate linkages were introduced by sulfurization with a 0.05 M solution of DDTT (3-((dimethylamino-methylidene)amino)-3H-1,2,4-dithiazole-3-thione) in 1:1 pyridine/CH_3_CN for a contact time of 3 min. Phosphate diester linkages were incorporated via oxidation of phosphite triesters using a solution of *tert*-butyl hydroperoxide/CH_3_CN/water (10:87:3) for a contact time of 12 min. All other steps in the protocol supplied by the manufacturer were used without modification. After the desired sequence was assembled, the solid-support bound oligonucleotide was washed with 1:1 TEA/CH_3_CN for 30 min. The solid-support bound oligonucleotide was suspended in aqueous ammonia (28–30 wt%) containing 1M 2-mercaptoethanol (used 1 mL/µmol of solid support) and either heated at 55 °C (**13**, Table 1) for 6 h or kept at room temperature for 24 h (**13**, Table 1) to complete cleavage from support. The unbound oligonucleotide was then filtered and the support was rinsed and filtered with water:ethanol (1:1) followed by water. The filtrate was combined and concentrated to dryness. The residue obtained was purified by HPLC (Gilson Inc., Middleton, WI, USA) on a reverse phase column (Waters X-Bridge C-18 5µm, 10 × 250 mm, A = 5 mM tributylammonium acetate in 5% aqueous CH_3_CN, B = CH_3_CN, 0 to 90% B in 80 min, flow 3 mL min^−1^, λ = 260 nm). Fractions containing full-length oligonucleotides were pooled together (assessed by LC/MS analysis > 95%) and the tributylammonium counter ion was exchanged to sodium by HPLC on a strong anion exchange column (GE Healthcare Bioscience, Pittsburgh, PA, USA) Source 30Q, 30 µm, 2.54 × 8 cm, A = 100 mM ammonium acetate in 30% aqueous CH_3_CN, B = 1.5 M NaBr in A, 0–40% of B in 60 min, flow 14 mL min^−1^). The residue was desalted by HPLC on reverse phase column to yield the oligonucleotide in an isolated yield of 15–20% based on solid-support loading. The oligonucleotides were characterized by ion-pair-HPLC-MS analysis with Agilent 1100 MSD system (Agilent Technologies, Santa Clara, CA, USA).

## 4. Conclusions

In this study, we designed and synthesized 5′-deoxy-(*E*)-5′-vinyl-phosphonate)-2′-*O*-(2-methoxyethyl)-thymidine-3′-phosphoramidite with a base-labile *t*Bu-SATE protected vinylphospho- nate in good yield. To examine the compatibility of the *t*Bu-SATE-protecting group under solid-phase oligonucleotide synthesis conditions, we synthesized a guide strand of fully modified siRNA containing (*E*)-5′-vinylphosphonate using this phosphoramidite. The *t*Bu-SATE-protected phosphor- amidite was successfully incorporated at the 5′-terminus of a 2′-F/2′-*O*-Me guide strand of siRNA using solid-phase DNA synthesis reagents and methods. Moreover, the *t*Bu-SATE group was conveniently removed under aqueous ammonia mediated deprotection conditions. The yield and purity of resulting oligonucleotides were identical to the one obtained using previously reported phosphoramidite containing vinylphosphonate methyl esters [13]. The strategy described in this report simplify the synthesis and deprotection of the siRNAs containing a metabolically stable (*E*)-5′-vinylphosphonate and eliminates the need for any additional steps or reagents.

## Figures and Tables

**Table 1:** Synthesis scale, deprotection condition and analytical data of guide strand **13** synthesized using *t*Bu-SATE vs. methyl protecting groups.

Comp.	Protecting Group	Deprotection Temperature	Mol. Wt. Calculated	Mol. Wt. Found	Scale μmol	%UV Purity
**13**	SATE	55 °C	7300.4	7299.3	2	99.3
**13**	SATE	25 °C	7300.4	7299.1	2	98.6
**13**	Me	55 °C	7300.4	7299.1	2	99.1
